# Aflatoxins Levels in Concentrate Feeds Collected from Specialized Dairy Farms and Local Markets in Selected Urban Centers of Eastern Ethiopia

**DOI:** 10.3390/toxins16100418

**Published:** 2024-09-27

**Authors:** Angassa Tesfaye, Mohammed Yusuf Kurtu, Yesihak Yusuf Mummed, Abdi Mohammed

**Affiliations:** 1 Department of Animal and Range Sciences, College of Agricultural Sciences, Bule Hora University, Bule Hora P.O. Box 144, Ethiopia; 2 School of Animal and Range Sciences, College of Agriculture and Environmental Sciences, Haramaya University, Dire Dawa P.O. Box 138, Ethiopia; 3 School of Plant Sciences, College of Agriculture and Environmental Sciences, Haramaya University, Dire Dawa P.O. Box 138, Ethiopia

**Keywords:** aflatoxins, feed and food safety, dairy farmers, feed retailers, maize feed, total mixed ration, wheat bran, HPLC/FLD, eastern Ethiopia

## Abstract

Aflatoxin constitutes a significant concern for food and feed safety, posing detrimental health risks to both animals and humans. This study aimed to examine the prevalence and concentration of aflatoxins in maize feed, total mixed ration, and wheat bran collected from specialized dairy farms and local markets in three major urban centers in eastern Ethiopia. A total of 180 feed samples were collected from September 2021 to January 2022 in Chiro town, Dire Dawa city, and Harar city. These samples underwent thorough extraction and immunoaffinity clean-up before aflatoxin analysis using HPLC/FLD. The results revealed that AFB_1_, AFB_2_, AFG_1_, AFG_2_, and TAF contamination was detected in 72.2%, 66.1%, 71.1%, 68.7%, and 82.8% of the feed samples, respectively. The corresponding mean levels of each aflatoxin were 28.15 ± 3.50, 3.3 ± 0.40, 19.87 ± 1.87, 2.7 ± 0.32, and 54.01 ± 4.72 µg/kg, respectively. The occurrence and levels of aflatoxin varied across different study sites and feed types. Notably, feeds from Dire Dawa city exhibited significantly higher mean levels of AFB_1_ (43.98 ± 5.3 µg/kg), AFB_2_ (5.69 ± 0.6 µg/kg), AFG_1_ (32.25 ± 2.7 µg/kg), and AFG_2_ (5.01 ± 0.5 µg/kg) than feeds from other urban centers did. Additionally, a significantly higher occurrence of AFB_1_ (29.4%) and AFG_1_ (28.3%) was detected in feed from Dire Dawa city. Similarly, the total mixed ration (TMR) displayed significantly higher levels of AFB_1_ (50.67 ± 5.2 µg/kg), AFB_2_ (4.74 ± 0.6 µg/kg), AFG_1_ (32.87 ± 2.6 µg/kg), and AFG_2_ (3.86 ± 0.5 µg/kg) compared to the other feed types. Moreover, a significantly higher occurrence of AFB_1_ (30.7%) and AFG_1_ (28.7%) was detected in the TMR. Furthermore, a moderate correlation was observed between the count of aflatoxigenic *Aspergillus* species and the levels of TAF in the feed samples. Overall, this study underscores the widespread presence of aflatoxin contamination in dairy feeds in eastern Ethiopia, highlighting the urgent need for stringent monitoring and mitigation measures to ensure food and feed safety, as well as public health.

## 1. Introduction

Aflatoxins are naturally occurring secondary metabolites, produced by *Aspergillus* species, primarily *A. flavus* and *A. parasiticus* [1]. Thus, *A. flavus* produces aflatoxin B_1_ (AFB_1_) and B_2_ (AFB_2_), while *A. parasiticus* secrets AFB_1_, AFB_2_, AFG_1_, and AFG_2_ [2,3]. *Aspergillus* fungi and subsequent aflatoxins are frequent contaminants of a wide variety of agricultural products, from the field to consumers’ plates [4]. Food and feed crops such as cereal grains, nuts, vegetables, fruits, and spices are frequently targeted by aflatoxins [5]. Thus, the structurally analogous aflatoxins, such as B_1_ (AFB_1_), B_2_ (AFB_2_), G_1_ (AFG_1_), and G_2_ (AFG_2_), are the major contaminants of animal feed and feed ingredients [2,3].

Exposure to aflatoxins poses a significant health risk to both humans and animals [6]. The International Agency for Research on Cancer (IARC) has classified aflatoxins as group 1 carcinogens [7,8]. The liver is the target organ, where chronic exposure of aflatoxin is associated with hepatocellular carcinoma (HCC) and other health complications [3,4]. On the other hand, acute aflatoxicosis can lead to liver disease and even death [4,9,10]. Consequently, investigations have demonstrated that thousands of human lives have been lost in numerous nations across the globe. Moreover, many millions of people in various countries are exposed to chronic aflatoxicosis, highlighting greater risks to feed and food safety and public health from aflatoxin [11,12].

Many countries have implemented stringent legal limits for aflatoxin levels in animal feeds and food products to minimize the risk of aflatoxins entering food chains. For instance, the European Commission has established a maximum tolerable limit of 20 µg/kg AFB_1_ in the feed for all animals and 5 µg/kg AFB_1_ in the feed for dairy cows [13]. The Ethiopian Standard Authority (ESA) set a maximum limit of 20 µg/kg for AFB_1_ and 40 µg/kg for total aflatoxin (TAF), whereas the Food and Drug Administration (FDA) set 20 µg/kg limit of TAF in dairy feed [14]. The implementation of strict regulations on aflatoxin levels in food and feed in developed countries has effectively reduced the risk of aflatoxin exposure [4]. However, in developing nations, the risk of aflatoxin exposure remains high due to insufficient regulations and implementation capacity.

Aflatoxin contamination in food and animal feeds is of particular concern in tropical and subtropical regions, where favorable climatic conditions promote the growth of aflatoxigenic *Aspergillus* fungi [15]. In addition, poor agricultural practices and crop damage prior to harvest significantly contribute to the increased risk of aflatoxin contamination in feed and feed ingredients [16]. Inadequate storage conditions such as air moisture, leakage of the floors/walls/roofs, and poor ventilation at dairy cattle farms and feed retailers also contribute to *Aspergillus* fungi and subsequent aflatoxin contamination in animal feed [17,18].

Contaminated feeds and feed ingredients are the main sources of aflatoxin exposure for animals. These contaminants then find their way into the food chain, primarily through milk and milk products, resulting in human contamination [19]. Aflatoxins frequently contaminate major crops such as maize, wheat, oilseeds, and others, which are the integral components of animal feeds [15]. Consequently, concentrate feeds such as wheat bran, oilseed cakes, maize-grain-based feed, and total mixed rations are the primary sources of aflatoxin contamination in animal and animal products [18,20,21].

Numerous investigations have demonstrated the prevalence of aflatoxin in dairy feeds. For instance, Nkosi et al. [22] reported that different dairy cattle feeds in Malawi had an average AFB_1_ concentration of 29.68 µg/kg and a contamination rate of 88.2% (*n* = 51), with 17.6% exceeding the 5 µg/kg standard set out by the EU. Similarly, AFB_1_ contamination was detected in 57% (*n* = 74) of concentrate feed samples in Kenya, with a mean level of 28.3 µg/kg, with 56% of the samples exceeding the standards set out by EU regulation [23]. In another study, a 48% contamination frequency and a mean level of 0.7 µg/kg AFB_1_, a 93% frequency and a mean level of 3.1 µg/kg AFB_2_, a 55% frequency and a mean level of 2.5 µg/kg AFG_1_, and a 100% frequency and a mean level of 41.3 µg/kg AFG_2_ were found in dairy feed from the Gauteng province of South Africa [24]. Additionally, Njobeh et al. [25] revealed that in South Africa, 14.7 µg/kg TAF and 52% (*n* = 25) prevalence were found in compound feed used for dairy cows, with 16% of samples exceeding the standards set out by EU regulation. Meanwhile, in Tanzania, a sunflower-based dairy feed had a prevalence of 72% (*n* = 18), with a higher mean level of 26 µg/kg for TAF [26].

In Ethiopia, like in most countries with tropical and subtropical climates, the presence of aflatoxin in dairy cattle feed poses significant concerns for food safety and public health [27,28]. Accordingly, numerous investigations have revealed substantial levels of aflatoxin contamination in dairy feed samples collected from various locations in Ethiopia. For instance, Gizachew et al. [29] reported a 100% contamination rate with a mean concentration of 362 ± 38 µg/kg in Noug cake, a 73% contamination rate with a mean concentration of 15 ± 6 µg/kg in wheat bran, and a 37% contamination rate with a mean concentration of 18 ± 11 µg/kg in maize grains for AFB_1_ in dairy feed collected from Addis Ababa, Ethiopia. Additionally, Rehrahie et al. [30] reported that 49.4% (n = 160) of dairy feed samples collected from various towns in central Ethiopia were contaminated with AFB_1_, with mean concentrations of 5.63 µg/kg, and 81.9% of the samples exceeded the FDA regulation limit of 20 µg/kg. Another study conducted by Yoseph et al. [31] reported a contamination rate of 31% (n = 33) with a mean concentration of 195.88 µg/kg, and 94% of the samples exceeded the FDA regulation limit for TAF in feed from Bishoftu town. The same authors reported mean concentrations of 17.93 µg/kg for AFB_1_, 91.37 µg/kg for AFB_2_, 13.79 µg/kg for AFG_1_, and 72.77 µg/kg for AFG_2_.

Furthermore, considering the rapid urbanization rate in Ethiopia and the significant role of specialized dairy farming in meeting milk demand in major urban centers [32,33], investigating the prevalence of aflatoxin in feed and milk is crucial. Therefore, this study focuses on concentrate feeds commonly used in specialized dairy farms and sold in local markets, such as wheat bran, maize feeds, and total mixed rations [34,35,36]. This study was conducted in three densely populated urban centers in eastern Ethiopia, which have many indoor dairy farms and feed retailers [37,38,39].

The objective of this study was to investigate the prevalence and concentration levels of aflatoxins in three types of feed collected from specialized dairy farms and local markets in Chiro town, Dire Dawa city, and Harar city in eastern Ethiopia. Additionally, this study aimed to evaluate the correlation between the counts of aflatoxigenic *Aspergillus* species and the levels of aflatoxins detected in the feed.

## 2. Results

### 2.1. Frequency of Occurrence of Aflatoxins in Dairy Feed

**Figure 1:**
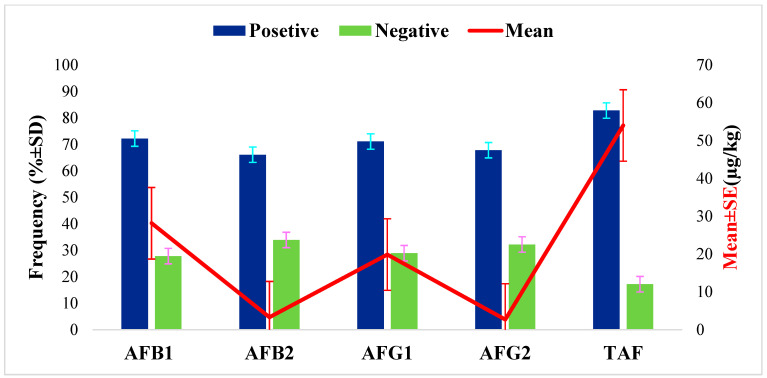
Overall frequency of occurrence and level of total aflatoxins in dairy feed (TAF = AFB_1_ + AFB_2_ + AFG_1_ + AFG_2_).

A total of 180 dairy cow feed samples were analyzed to quantify AFs using HPLC following validation of the measurement procedures as described in the methodology. The results revealed that 82.80% of samples (149/180) were contaminated with at least one AF, with an average concentration of 54.01 ± 4.72 µg/kg (Figure 1). The results also showed that all types of aflatoxin were highly prevalent, with 72.2%, 71.1%, 67.8% and 66.1% of feed samples being contaminated with AFB_1_, AFG_1_, AFB_2_, and AFG_2_, respectively, with mean concentrations of 28.15 ± 3.50, 19.87 ± 1.87, 3.30 ± 0.40, and 2.70 ± 0.32 µg/kg, respectively (Figure 1). However, in 17.2%, 27.8%, 33.9%, 28.9%, and 32.2% of the feed samples, TAF, AFB_1_, AFB_2_, AFG_1_, and AFG_2_, respectively, were not detectable.

The analysis of variance showed that there was significant variation in the mean concentration of TAF among study sites (*p* < 0.01). Thus, a significantly higher (*p* < 0.05) mean concentration of TAF was detected in feed samples collected from Dire Dawa city (mean = 86.93 ± 8.2 µg/kg; range = ND–455.8 µg/kg) than in those from Harar city (mean = 41.50 ± 8.2 µg/kg; range = ND–229.2 µg/kg) and Chiro town (mean = 33.60 ± 8.2 µg/kg; range = 150.6 µg/kg). Similarly, the ANOVA result showed a highly significant variation in the mean concentration of TAF among the examined feed types, with TMR having a significantly (*p* < 0.01) higher mean level of TAF (mean = 92.20 ± 8.2 µg/kg; range = ND) compared to MF (mean = 43.80 ± 8.2 µg/kg; range = ND–175.9 µg/kg) and WB (mean = 26.0 ± 8.2 µg/kg; range = ND–245.5 µg/kg). However, no variation was observed among the sources of feed samples in the mean concentration of TAF (Table 1).

The results also revealed that the prevalence of feed samples contaminated with total aflatoxin did not vary (*p* > 0.05) across study sites, feed sources, and feed types (Table 1). However, a proportionally higher frequency of occurrence of TAF was found in the feed samples collected from Dire Dawa city (29.4%, n = 53) compared to the feed collected from Harar city (27.2% n = 49) and Chiro town (26.1%, n = 47). Likewise, a numerically higher frequency of occurrence of TAF was observed in TMR (30.0%, n = 54) than in MF (27.8%, n = 27.8) and WB (25.0%, n = 45). Moreover, a relatively similar frequency of occurrence of TAF was observed in samples from dairy farms (41.7%, n = 75) and local markets (41.1%, n = 74) (Table 1).

### 2.2. Frequency of Occurrence and Level of Aflatoxins in Feed across Study Sites, Feed Sources, and Feed Types

The analysis of variance showed that the mean concentration of AFB_1_ recovered from the dairy feeds using HPLC/FLD significantly varied among study sites and feed types (*p* < 0.001) (Table 2), as well as revealing an interaction between feed types and study sites (*p* < 0.05) (Table 2). However, variation in mean concentrations of AFB_1_ was not observed within feed sources across all study sites. Among the study sites, the highest mean concentration of AFB_1_ was recovered in the samples collected from Dire Dawa city (mean = 43.98 ± 5.3 µg/kg; range = LOD−303.5 µg/kg) compared to the other urban centers. On the other hand, among feed types, TMR had the highest significant (*p* < 0.05) mean concentration of AFB_1_ (mean = 50.67 ± 5.2 µg/kg; range = LOD−303.5 µg/kg) compared to the other feed types (Table 2). Moreover, the interaction in the mean concentration of AFB_1_ was observed to be significantly *(p* < 0.05) different among feed types and study sites. Thus, in Chiro town, the lowest significant (*p* < 0.05) mean concentration of AFB_1_ was observed in WB (6.6 ± 8.9 µg/kg) compared to the MF (29.8 ± 8.9 µg/kg) and TMR (18.7 ± 8.9 µg/kg), respectively. However, in Dire Dawa city and Harar city, the highest significant (*p* < 0.05) mean concentration of 94.9 ± 8.9 µg/kg and 38.4 ± 8.9 µg/kg for AFB_1_ was detected in TMR, respectively, compared to other feed types (Table 3).

The result of ANOVA also revealed that the mean concentration of AFB_2_ observed in this study varied significantly among the study sites (*p* < 0.001) and feed types (*p* < 0.05), while non-significant (*p* > 0.05) variation was observed between feed sources (Table 2). In comparison, the highest significant (*p* < 0.05) mean concentration of AFB_2_ was detected in feed samples collected from Dire Dawa city (mean = 5.69 ± 0.6 µg/kg; range = LOD–30.3 µg/kg) (Table 2). Among the feed types from both sources, the highest significant (*p* < 0.01) mean concentration of AFB_2_ was detected in TMR (mean = 4.74 ± 0.6 µg/kg, range = LOD–25.3 µg/kg) collected from all study sites (Table 2).

Furthermore, this study shows that the prevalence of AFB_1_ and AFB_2_ in feed samples varied across study sites, feed sources, and feed types (Table 2). Thus, the proportion of dairy feed contaminated with AFB_1_ (29.4%, n = 53/180) in feed samples from Dire Dawa city was significantly (*p* < 0.05) higher compared to the samples from Chiro town (22.3%, n = 40/180) and Harar city (20.7%, n = 37/180). Moreover, a numerically higher frequency of occurrence of AFB_1_ was observed in feed samples from dairy farms (37.2%, n = 67/180) than in the feed collected from markets (35.0%, n = 63/180). However, the frequency of occurrence of AFB_2_ did not significantly (*p* < 0.05) differ across the study sites, feed sources, and feed types. But the numerically highest occurrence of AFB_1_ was observed in feed samples from Dire Dawa city (25.0%, n = 45/180) and dairy farms (37.2%, n = 67/180), as well as in the TMR feed type (26.1%, n = 47/180) (Table 2).

In addition, the analysis of variance revealed that the mean level of AFG_1_ recovered from the examined feed samples significantly varied among the study sites and feed types (*p* < 0.01) (Table 4), as well as among feed types and the study sites interaction (*p* < 0.05) (Table 4). However, variation in the mean concentration of AFG_1_ was not observed within the feed sources across all sites. Regarding the study sites, the highest significant (*p* < 0.001) mean concentration of AFG_1_ was recovered in the feed samples collected from Dire Dawa city (mean = 32.25 ± 2.7 µg/kg; range = LOD–125.5 µg/kg) compared to the other urban centers (Table 4). Similarly, among feed types, TMR had a significantly (*p* < 0.01) higher mean concentration of AFG_1_ (mean = 32.87 ± 2.6; range = LOD−125.5) than the other feed types (Table 4). However, the variation in the mean concentration of AFB_1_ observed among feed types was not the same across the urban centers. As a result, in Dire Dawa city and Harar city, the TMR feed samples had a significantly (*p* < 0.001) higher mean concentration of 60.0 ± 4.6 µg/kg and 24.3 ± 4.6 µg/kg for AFG_1_, respectively, compared to the other feed types. However, in Chiro town, a non-significant (*p* > 0.05) mean concentration of AFG_1_ was observed between all feed type (Table 3).

The result of ANOVA also revealed that the mean concentration of AFG_2_ observed in this study varied significantly among study sites (*p* < 0.01) and feed types (*p* < 0.01), while there was no significance (*p* > 0.05) observed between feed sources (Table 4). In comparison, a significantly higher mean concentration of AFG_2_ was detected in feed samples collected from Dire Dawa city (mean = 5.01 ± 0.5 µg/kg; range = LOD–21.5 µg/kg) compared to the feed samples from Chiro town (mean = 1.67 ± 0.5 µg/kg; range = LOD–9.4 µg/kg) and Harar city (mean = 1.37 ± 0.5 µg/kg; range = LOD–9.8 µg/kg) (Table 4). Similarly, among the examined feed types, the highest average concentration of AFG_2_ was detected in TMR feed samples (mean 3.86 ± 0.5 µg/kg; range = LOD–21.5 µg/kg) compared to the MF (mean = 2.60 ± 0.5 µg/kg; range = LOD–11.5 µg/kg) and WB (mean = 1.58 ± 0.5 µg/kg; range = LOD–21.5 µg/kg) collected from all study sites (Table 4).

The study results revealed that the prevalence of AFG_1_ and AFG_2_ in feed samples was observed at varying proportions across the study sites, feed sources, and feed types (Table 4). Thus, the proportion of dairy feed contaminated with AFG_1_ (28.3%, n = 51/180) in feed samples from Dire Dawa city was significantly (*p* < 0.05) higher compared to the proportion in feed samples from Chiro town (22.8%, n = 41/180) and Harar city (20.0%, n = 36/180). Moreover, the highest (*p* < 0.05) occurrence of AFG_1_ was observed in TMR (28.7%) compared to MF (21.9%) and WB (20.7%). However, a numerically higher occurrence of AFG_1_ was observed in feed samples from dairy farms (36.1%, n = 65/180) than in the feed collected from local markets (35.0%, n = 63/180). On the other hand, the frequency of occurrence of AFG_2_ did not significantly (*p* < 0.05) differ across study sites, feed sources, and feed types. However, a numerically higher frequency of occurrence of AFG_2_ was observed in feed samples from Dire Dawa city (25.6%, n = 46/180), dairy farms (35.6%, n = 64/180), and in the TMR feed type (23.3%, n = 42/180) (Table 4).

### 2.3. Level of Aflatoxins in Feeds beyond Different Regulatory Limits

Furthermore, the proportion of feed samples contaminated with TAF and AFB_1_ exceeding the limits set by the FDA/ESA is presented in Table 5. Overall, 33.3% and 38.9% of feed samples were found to exceed the 20.0 µg/kg regulation set by the FDA/ESA for AFB_1_ and the 40.0 µg/kg regulation set by the ESA for TAF, respectively. Moreover, a higher proportion of feed samples exceeding the ESA regulation for TAF was observed in the samples collected from Dire Dawa city (56.7%) compared to those from Chiro town (26.7%) and Harar city (33.3%). Similarly, a higher proportion of feed samples with an AFB_1_ content exceeding the FDA/ESA limit was found in the samples from Dire Dawa city (41.7%) compared to those from Chiro town (25.0%) and Harar city (33.3%).

On the other hand, a higher proportion of feed samples exceeding the ESA limit for TAF (43.3%), as well as feed samples exceeding the FDA/ESA regulation for AFB_1_ (42.2%), was found in samples from specialized dairy farms compared to those from local markets. Furthermore, among the analyzed feed types, a higher proportion of feed samples exceeding the ESA regulation limit for TAF (53.3%) and the FDA/ESA regulation limit for AFB_1_ (50.0%) was found in TMR. Meanwhile, 43.3% of MF and 20.0% of WB feed samples containing TAF exceeded the ESA regulation limit, while 33.3% of MF and 16.7% of WB feed samples containing AFB_1_ exceeded the FDA/ESA regulation limit.

### 2.4. Correlations between Aflatoxigenic *Aspergillus* Isolates and Aflatoxins Level

**Figure 2:**
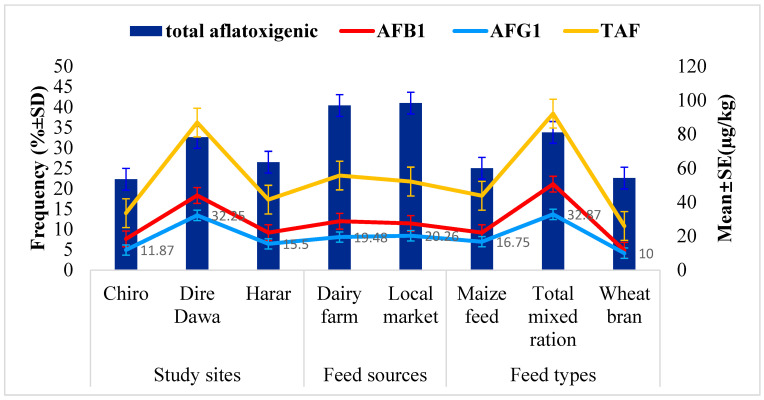
Relationship between the contamination of aflatoxigenic *Aspergillus* species isolates and the level of TAF, AFB_1_, and AFG_1_.

The relationship between the occurrence of aflatoxigenic *Aspergillus* species isolates and the TAF, AFB_1_ and AFG_1_ levels in feed samples across study sites, feed types, and feed sources is presented in Figure 2. Comparably, the highest occurrence of aflatoxigenic *Aspergillus* species isolates (32.6%) was found in feed samples from Dire Dawa city compared to those from the other urban centers. Similarly, the corresponding mean levels of 86.93 µg/kg for TAF, 43.98 µg/kg for AFB_1_ and 32.25 µg/kg AFG_1_ in the feed samples from Dire Dawa city were found to be higher than in those from the other study sites. The results revealed that the TMR feed type had the highest proportion of aflatoxigenic *Aspergillus* species (33.8%), as well as the highest mean levels of TAF (92.2 µg/kg), AFB_1_ (50.67 µg/kg), and AFG_1_ (32.87 µg/kg).

On the other hand, comparable aflatoxigenic *Aspergillus* species contamination rates in the feed from dairy farms (40.4%) and local markets (41.0%) were observed. In line with this, comparable mean concentrations of TAF (55.7 µg/kg), AFB_1_ (28.81 µg/kg), and AFG_1_ (19.48 µg/kg) were observed in feed samples from dairy farms compared to the corresponding mean concentrations of 52.2 µg/kg, 27.48 µg/kg, and 20.26 µg/kg for the corresponding aflatoxins in feed from local markets.

Furthermore, Table 6 presents the correlation between the aflatoxigenic *Aspergillus* species isolates and the aflatoxin concentration levels in feed samples. Thus, the isolates of aflatoxigenic *A. flavus* showed a moderate correlation (*p* < 0.01) with AFB_1_ (r = 0.47) and AFB_2_ (r = 0.46). Similarly, the isolates of aflatoxigenic *A. parasiticus* showed a moderate correlation (*p* < 0.01) with AFB_1_ (r = 0.43), AFB_2_ (r = 0.40), AFG_1_ (r = 0.50), and AFG_2_ (r = 0.39). Overall, the correlation analysis revealed a moderately positive correlation between the counts of aflatoxigenic *Aspergillus* species and the concentrations of aflatoxins in the feeds of dairy cows. This confirms that the identified aflatoxigenic *Aspergillus* isolates were responsible for the contamination of aflatoxins in the examined dairy feeds. However, a correlation analysis of aflatoxigenic *A. flavus* revealed non-significant correlation (*p* > 0.05) with the concentration of AFG_1_ and AFG_2_, confirming the fact that not all aflatoxins were produced by *A. flavus*.

## 3. Discussion

### 3.1. Occurrence and Levels of Aflatoxins in Feed

The persistent occurrence of aflatoxins in food and feed has detrimental effects on human and animal health. Aflatoxin contamination in feed for dairy cattle can then be transferred to humans via the consumption of products from animals that have been exposed to contaminated feed [19]. Therefore, it is essential to assess the prevalence of aflatoxins in dairy cow feed that is prone to aflatoxin contamination to understand the associated public health risks and thereby plan for their prevention and control. As a result, in this study, 180 samples of the three most susceptible feed types for dairy cows were collected from specialized dairy farms and local markets in three selected urban centers in eastern Ethiopia and examined for AFB_1_, AFB_2_, AFG_1_, and AFG_2_ contamination.

In the present study, among all the tested samples (N = 180), about 82.8% were contaminated with a mean level of 54.01 ± 4.62 µg/kg total aflatoxin (TAF) in dairy feeds, which is higher than the previous levels reported in Ethiopia by Mulugeta [40] and Yohannes et al. [41]. Contamination levels of 50.0%, with a mean concentration of 0.69 µg/kg in dairy feed collected from dairy farmers and feed traders around Addis Ababa, and 52.7%, with a mean concentration of 10.54 ± 3.82 µg/kg in dairy feed from the Gurage zone were reported in these studies [40,41]. However, a higher mean level of TAF in dairy feed (313.03 µg/kg) and Niger seed cake feed (385.45 µg/kg) collected from Addis Ababa, Ethiopia was reported [42]. Similarly, a study conducted in Bishoftu town in Ethiopia revealed a higher level of TAF (195.88 µg/kg) in poultry feed compared to our findings [31].

Additionally, as shown in Table 2, the mean level of TAF in the feed samples varied among the different study sites, feed sources, and feed types. Consequently, the feed samples collected from Dire Dawa city showed a significantly higher mean concentration of 86.93 ± 8.2 µg/kg for TAF. In contrast, the mean concentration of 41.5 ± 8.2 µg/kg for TAF in the feed samples from Harar city was not statistically significant compared to the 33.6 ± 8.2 µg/kg mean concentration of TAF in the feed samples from Chiro town. In a study conducted by Yohannes et al. [41], a comparably lower mean of 10.54 ± 3.82 µg/kg for TAF in the feeds from Butajira town and 4.22 ± 8.2 µg/kg for TAF in feed from Emdibir town in the Gurage Zone, Ethiopia was detected. However, there was a higher mean of 313.03 µg/kg for TAF in dairy feed collected from feed factories in Addis Ababa, Ethiopia. Furthermore, poultry feed collected from different sites in Bishoftu town in Ethiopia had a higher mean TAF concentration (195.88 µg/kg) [31] compared with the present findings. These variations in aflatoxin levels may be attributed to various factors, including geographical location, climatic conditions, and feed storage practices [15,43,44]. In particular, poor storage practices among dairy farmers, such as inadequate air flow and ventilation, elevated air moisture and temperature, and prolonged storage period may have contributed to the proliferation of *Aspergillus* fungi and the production of aflatoxin.

Similarly, TMR had a significantly higher mean level of TAF (92.2 ± 8.2 µg/kg) compared to the other feed types. However, there was no significant difference between the average levels of TAF in maize feeds (43.8 ± 8.2 µg/kg) and those in wheat bran (26 ± 8.2 µg/kg). In a previous study, Genet et al. [42] reported a mean level of 385.45 µg/kg for TAF in Noug seed cake (*Guizotia abyssinica*), which was higher than the mean level of 26.92 µg/kg in maize and 3.7 µg/kg in wheat bran collected from Addis Ababa, Ethiopia. Furthermore, Mulugeta [40] reported a lower mean level of 0.06 µg/kg for TAF in wheat bran collected from different towns in central Ethiopia. The variations in aflatoxin levels in different types of feed could be due to the presence of susceptible feed ingredients such as Noug seed cake [6], which is a crucial ingredient in TMR. Consistent with this finding, Gizachew et al. [29] reported that Noug seed cake had the highest concentration of aflatoxin and noted it as a highly susceptible feed ingredient to aflatoxin contamination.

Our results also revealed high rates of contamination with AFB_1_, AFB_2_, AFG_1_, and AFG_2_ among the examined dairy feeds. Thus, this investigation demonstrated a common pattern of aflatoxin occurrence, with higher amounts of the highly potent AFB_1_ and AFG_1_ than AFB_2_ and AFG_2_. As a result, AFB_1_ was detected in 72.2% of samples, with a mean level of 28.15 ± 3.50 µg/kg, while AFG_1_ was detected in 71.1% of samples, with a mean level of 19.87 ± 1.87 µg/kg. In comparison, AFB_2_ had an occurrence rate of 66.1% with a mean level of 3.30 ± 0.40 µg/kg, whereas AFG_2_ had an occurrence rate of 67.8% with a mean level of 2.70 ± 0.32 µg/kg. Similar to our findings, higher levels of AFB_1_ (31.2 µg/kg) and G_1_ ((17.2 µg/kg) relative to AFB_2_ (3.27 µg/kg) and AFG_2_ (1.14 µg/kg) have been reported in dairy feed from the Guraghe Zone in Ethiopia [41]. Similarly, Genet et al. [42] reported higher levels of AFB_1_ (192.8 µg/kg) and AFG_1_ (104.35 µg/kg) compared to the levels of AFB_2_ (12.34 µg/kg) and AFG_2_ (3.55 µg/kg) in dairy feed, as well as higher level of AFB_1_ (288.34 µg/kg) and AFG_1_ (82.9 µg/kg) compared to AFB_2_ (10.91 µg/kg) and AFG_2_ (3.28 µg/kg) in maize feed from Addis Ababa, Ethiopia. However, Yoseph et al. [31] reported higher levels of AFB_2_ (91.37 µg/kg) and AFG_2_ (72.77 µg/kg) than the levels of AFB_1_ (17.93 µg/kg) and AFG_1_ (13.79 µg/kg) in poultry feed from Bishoftu town, Ethiopia.

These variations may be attributed to multiple factors that affect the growth of aflatoxin-producing fungi and their potential for aflatoxin production. AFB_1_ and AFB_2_ are known to be produced by *A. flavus,* whereas *A. parasiticus* produces all distinct types of aflatoxins B_1_, B_2_, G_1_, and G_2_ [45,46,47]. Moreover, another investigation demonstrated that the ratio of B and G aflatoxin is largely influenced by the conditions within the ecological niches where fungal species that produce aflatoxins grow [15]. Predominantly, temperature plays a greater role in AFB_1_ production, whereas water activity is vital for AFG_1_ biosynthesis [48]. Moreover, Medina et al. [49] noted that both water availability and temperature affect the expression of the structural gene (*aflS*) and regulatory gene (*aflR*), which determine the relative growth and aflatoxin production in both *A. flavus* and *A. parasiticus*. Similarly, a study by Abdel-Hadi et al. [50] revealed a good correlation between the early structural gene and aflatoxin production. In addition, Medina et al. [49] reported the interaction of conditions with relative gene expression and AFB_1_ production, involving temperature, water activity, and other factors.

### 3.2. Mean Concentration and Occurrence of Principal Aflatoxins in Feeds

The mean concentrations and frequency of occurrence of AFB_1_, AFB_2_, AFG_1_, and AFG_2_ in the feed samples were compared across the study sites, feed sources, and feed types (Table 2 and Table 4). Thus, significantly (*p* < 0.001) different mean concentrations of AFB_1_, AFB_2_, AFG_1_ and AFG_2_, as well as a significantly (*p* < 0.05) different frequency of occurrence of AFB_1_ and AFG_1_, were observed between the study sites. As a result, a significantly (*p* < 0.001) higher mean concentration of 43.98 ± 5.3 µg/kg for AFB_1_, with an occurrence rate of 29.4%, and a mean concentration of 32.25 ± 2.7 µg/kg for AFG_1_, with an occurrence rate of 28.3%, were found in feed samples collected from Dire Dawa city compared to the other urban centers. Similarly, the mean levels of AFB_2_ (5.69 ± 0.6 µg/kg) and AFG_2_ (5.01 ± 0.5 µg/kg) in the feed samples from Dire Dawa city were significantly (*p* < 0.01) higher compared to the other study sites. Since the feed types analyzed across the study sites were the same, the differences in climatic conditions, feed storage practices, and moisture content might be the cause of the variation in aflatoxin contamination in the feeds between the study sites.

In accordance with the current findings, Jalel et al. [18] reported a significant (*p* < 0.0001) mean concentration of AFB_1_ in dairy feed from Sululta town (23.37 ± 1.95 µg/kg) compared to Burayu town (21.99 ± 1.75 µg/kg) and Sebeta town (21.9 ± 1.9 µg/kg). Similarly, Gizachew et al. [29] reported significantly different levels of aflatoxin in feed between different study towns. Thus, the feed samples from Debre Zeit town had over three times higher levels of AFB_1_ compared to the feeds from Sendafa, Sululta, and Addis Ababa. Consistent with our findings, Jalel et al. [18] and Gizachew et al. [29] noted that environmental temperature, feed moisture content, and storage situation were the contributing factors to the variation in aflatoxins between the study areas. In contrast, Mulugeta, [40] reported that the levels of aflatoxins B_1_, B_2_, G_1_ and G_2_ in dairy feeds were not significantly different across three different towns (Sululta, Bishoftu, and Debre Berhan) in central Ethiopia.

Moreover, Rehrahie et al. [30] reported a lower mean level of AFB_1_ (5.63 µg/kg) in dairy feed from different towns in central Ethiopia compared to the present study, with a higher frequency of occurrence (49.4%). Compared with the present study, Changwa et al. [24] reported lower levels of AFB_1_ (0.7 µg/kg) and AFG_1_ (2.6 µg/kg) but higher levels of AFG_2_ (41.3 µg/kg) in dairy feeds from South Africa. However, the same authors reported a higher occurrence of AFB_1_ (48.0%), AFB_2_ (93.0%), AFG_1_ (55.0%) and AFG_2_ (100.0%) in dairy feeds compared with the present study. On the other hand, Genet et al. [42] reported higher mean concentrations of AFB_1_ (192.80 µg/kg), AFB_2_ (12.34 µg/kg), and AFG_1_ (104.35 µg/kg) in dairy feed collected from Addis Ababa compared to the present study.

In line with this finding, several studies have revealed that the variation in the level of aflatoxins across different study areas may be attributed to geographical locations, climatic conditions, and feed storage practices or a combination of these factors under which the *Aspergillus* fungi that produce aflatoxin grow [15,51,52]. *A. flavus* is widely known to produce AFB_1_ and AFB_2_, whereas *A. parasiticus* produces all distinct types of aflatoxin, including B_1_, B_2_, G_1_, and G_2_ [45,46,47]. According to Matumba et al. [48], temperature plays a greater role in AFB_1_ production, whereas water activity/moisture content contributes to the biosynthesis of AFG_1_.

The study results revealed that significantly different (*p* < 0.01) mean concentrations of AFB_1_, AFB_2_, AFG_1_ and AFG_2_, as well as a significantly (*p* < 0.05) different frequency of occurrence of AFB_1_ and AFG_1_, were observed among the examined feed types (Table 2 and Table 4). Compared with the other feed types, the TMR feed exhibited a significantly higher mean concentration of 50.67 ± 5.2 µg/kg (LOD–303.5 µg/kg) and a 30.7% frequency of occurrence for AFB_1_, as well as a 32.87 ± 2.6 µg/kg (LOD–125.5 µg/kg) mean concentration and 28.7% frequency of occurrence for AFG_1_. Similarly, the average levels of 4.74 ± 0.6 µg/kg (LOD–25.3 µg/kg) for AFB_2_ and 3.86 ± 0.5 µg/kg (LOD–25.3 µg/kg) for AFG_2_ in TMR were significantly higher than those in the other feed types, although their frequency of occurrence did not significantly differ between feed types.

In line with this finding, Makau et al. [53] reported a highly significant (*p* < 0.001) variation in the mean concentration of AFB_1_ in different feed types, with a higher mean level detected in the mixed concentrate feed (147.86 µg/kg) than in the other feed types. Similarly, Mulugeta et al. [40] reported various mean concentrations of AFB_1_ (0.34 µg/kg), AFB_2_ (0.22 µg/kg), AFG_1_ (0.03 µg/kg), AFG_2_ (0.33 µg/kg), and TAF (0.61 µg/kg) in Noug seed cake compared with the corresponding mean levels of 0.021 µg/kg, 0.018 µg/kg, LOD µg/kg, 0.018 µg/kg, and 0.06 µg/kg in wheat bran collected around Addis Ababa, Ethiopia.

Moreover, research has revealed varied levels of aflatoxins in different feed types of dairy cows. Thus, compared with the present findings, lower mean levels of AFB_1_ (31.2 µg/kg) and AFG_1_ (17.1 µg/kg) were detected in the mixed feed of dairy cattle [41]. The same authors also reported comparable mean levels of AFB_2_ (3.27 µg/kg) and AFG_2_ (1.14 µg/kg) in mixed feed of dairy cows. Similarly, Genet et al. [42] reported lower concentrations of AFB_1_ (12.71 µg/kg), AFB_2_ (1.32 µg/kg), AFG_1_ (11.54 µg/kg), and AFG_2_ (1.35 µg/kg) in maize feed collected from Addis Ababa. In contrast, higher average levels of AFB_1_ (40.56 ± 9.58 µg/kg) were detected in maize bran from Malawi [22], whereas a comparable mean level of AFB_1_ (18 ± 11.0 µg/kg) was detected in maize grain collected from dairy farms from greater Addis Ababa milk shed, Ethiopia [29].

Additionally, in wheat bran from around Addis Ababa in Ethiopia, the mean concentrations of AFB_1_ (0.021 µg/kg), AFB_2_ (0.018 µg/kg), AFG_1_ (0.187 µg/kg), and AFG_2_ (0.0 µg/kg) were lower than in our findings [40]. Similarly, wheat bran collected from Addis Ababa feed factories had lower mean concentrations of AFB_1_ (2.29 µg/kg) and AFG_1_ (1.5 µg/kg) [42]. However, Gizachew et al. [29] reported a higher mean concentration of 15 ± 6 µg/kg for AFB_1_ in wheat bran collected from dairy farms in Addis Ababa city and the surrounding areas. Compared with the presented study, Elzupir et al. [54] reported higher mean concentrations of AFB_1_ (21.84 ± 0.7 µg/kg), AFB_2_ (5.03 ± 0.52 µg/kg), AFG_1_ (23.49 ± 0.75 µg/kg), and AFG_2_ (38.41 ± 0.41 µg/kg) in wheat bran from Khartoum, Sudan.

In addition to environmental factors, the type of substrate, nutrient composition, and moisture content of feed may play critical roles in the variation in the content of aflatoxin-producing fungi as well as aflatoxin levels between the examined feed types. In line with this finding, Kos et al. [55] noted that the degree of colonization by *Aspergillus* fungi in a given food or feedstuff depends on numerous factors, including the composition of the substrate, the availability of nutrients, moisture content, among others. Moreover, Daou et al. [56] noted that fungi may grow quickly on a substrate that contains high levels of carbohydrates and is rich in carbon and nitrogen. Thus, this may hold true in our cases, where the TMR (13.0%) has a greater level of crude fiber than the MF (2.2%) and WB (8.2%) do [57].

Furthermore, a significant (*p* < 0.001) interaction effect on the mean concentrations of AFB_1_ and AFG_1_ was observed among different feed types and study sites (Table 3). Thus, in Dire Dawa city and Harar city, the significantly (*p* < 0.001) highest mean concentrations of 94.908 ± 8.9 µg/kg and 38.4 ± 8.9 µg/kg for AFB_1_, respectively, were detected in TMR feed, whereas in Chiro town, the significantly (*p* < 0.001) lowest mean concentration of AFB_1_ was detected in the WB (6.6 ± 8.9 µg/kg). Similarly, the TMR from Dire Dawa city (60.0 ± 4.6 µg/kg) and Harar city (24.3 ± 4.6 µg/kg) had significantly (*p* < 0.001) higher mean concentrations of AFG_1_ than the other feed types. The significant variation in mean concentrations of AB_1_ and AFG_1_ in TMR feed samples from Dire Dawa city and Harar city may be attributed to a combined effect of relatively higher temperatures in these urban centers, as well as high carbohydrate (13.0%) and moisture content (11.8%) in TMR feed [58], which promote *Aspergillus* fungal growth and aflatoxin production. In line with this finding, Matumba et al. [48] noted that higher environmental temperature plays a greater role in AFB_1_ production, whereas moisture content contributes more for the biosynthesis of AFG_1_ in *Aspergillus* fungi.

### 3.3. Level of Aflatoxins in Feeds beyond Different Standard Regulations

The proportions of feed samples contaminated with AFB_1_ and TAF at levels exceeding the regulatory limits set by the FDA/ESA were determined across the study sites, feed sources, and feed types (Table 5). As a result, 33.3% (60/180) of the feed samples contaminated with AFB_1_ displayed levels exceeding the FDA/ESA limit of 20.0 µg/kg for dairy cattle feed. However, 38.9% (70/180) of the feed samples contaminated with TAF displayed levels exceeding the ESA limit of 40.0 µg/kg. A study conducted by Rehrahie et al. [30] revealed that 81.9% of feed samples exceeded the AFB_1_ level set out by the FDA/ESA regulation limit in dairy feed, which was higher than the proportion reported in the present study. Similarly, studies conducted on dairy feed collected from Nakuru County, Kenya [53] and Qazvin, Iran [59] revealed that 52.0% and 50.0% of feed samples containing AFB_1_ displayed levels exceeding the FDA regulatory limit, respectively. However, Jalel et al. [18] reported that 26.7% of feed samples contained AFB_1_ at concentrations exceeding 20.0 µg/kg.

On the other hand, a relatively higher percentage (62.5%) of feed samples with TAF above the FDA limit in dairy feeds has been reported [24]. The discrepancy in the proportion of feed samples with aflatoxins exceeding different regulatory limits may be attributed to geographical location, climatic conditions, or other factors favorable for aflatoxin production by *Aspergillus* fungus [15].

### 3.4. Correlation of Aflatoxigenic *Aspergillus* Isolates and Aflatoxins Level

Figure 2 presents the correlation of total aflatoxigenic *Aspergillus* isolates and the levels of TAF, AFB_1_, and AFG_1_ detected in the examined dairy feed samples. The highest occurrence of total aflatoxigenic *Aspergillus* species isolates (32.6%) was found in the feed samples from Dire Dawa city, with corresponding mean levels of 43.98 µg/kg for AFB_1_, 32.25 µg/kg for AFG_1_, and 86.93 µg/kg for TAF, which were also higher in the feed samples from Dire Dawa city. Similarly, the highest proportion of total aflatoxigenic *Aspergillus* species isolates (33.8%) was found in the TMR feed samples, with corresponding mean levels of 92.2 µg/kg for TAF, 32.87 µg/kg for AFG_1_, and 50.37 µg/kg for AFB_1_.

Although data on the relationship between aflatoxigenic *Aspergillus* species and aflatoxin levels are difficult to find, Krnjaja et al. [60] reported a comparable aflatoxigenic *Aspergillus* species occurrence rate of 85.71%, with a corresponding mean level of 4.47 µg/kg (1.79–16.01 µg/kg) in chicken feed, as well as a 100.0% occurrence rate with corresponding mean levels of 4.56 µg/kg (1.34–18.29 µg/kg) in layer feed for AFB_1_. Similarly, the highest occurrence of aflatoxigenic *A. flavus* in concentrate feed with a mean of 11.5 ± 8.0 µg/kg (2.6–24.8 µg/kg) for AFB_1_ was found compared to the other feed types of dairy cattle [61].

Moreover, Table 6 displays the Pearson correlation between the counts of aflatoxigenic *A. flavus* and *A. parasiticus* and the levels of aflatoxins. A highly significant (*p* < 0.01) moderate correlation was observed between AFB_1_ and AFB_2_ and aflatoxigenic *A. flavus*. A similar correlation was observed between aflatoxigenic *A. parasiticus* counts and the levels of AFB_1_, AFB_2_, AFG_1_, and AFG_2_ in dairy feed. Similarly, Kim et al. [60] reported a moderately positive correlation (r = 0.41) between the total fungal counts and the levels of AFB_1_ in poultry feed. However, a strong positive correlation (r = 0.76) was reported between the isolates of *A. flavus* in feed and the level of AFM_1_ in milk [11].

## 4. Conclusions

The investigation of aflatoxin prevalence in feed samples revealed significant occurrences and mean concentrations across study sites, feed sources, and feed types. Among the examined samples, an 82.8% prevalence with a mean level of 54.01 ± 4.62 µg/kg for TAF was found. Moreover, a higher occurrence of 72.2% with a mean level of 28.15 ± 3.50 µg/kg for AFB_1_ and an occurrence of 71.1% with a mean level of 19.87 ± 1.87 µg/kg for AFG_1_ were detected. Furthermore, the feed samples collected from Dire Dawa city exhibited a significantly higher prevalence and mean concentrations of highly potent AFB_1_ and AFG_1_ compared to the feed from Chiro town and Harar city. This could be attributed to suitable climatic factors for fungal colonization and aflatoxin production in feed samples from Dire Dawa city. Similarly, compared to MF and WB feed samples, the TMR feed samples showed a significantly higher prevalence and mean concentrations of the highly potent AFB_1_ and AFG_1_. This could be attributed to the nutrient composition and relatively favorable moisture content of TMR for *Aspergillus* fungal contamination and aflatoxin production.

In addition, a considerable proportion of the feed samples exceeded the FDA/ESA regulatory limits for AFB_1_ and TAF, particularly among the TMR feed samples. Moreover, the proportions of feed samples from specialized dairy farms that surpassed the FDA/ESA regulatory limits were higher than those from local markets. Moreover, there was a moderately positive correlation between aflatoxigenic *Aspergillus* species counts and the levels of TAF, indicating the aflatoxin production potential of *Aspergillus* species in feeds from eastern Ethiopia. This study revealed the widespread prevalence of aflatoxin contamination in dairy feed from the study areas. Thus, there is an urgent need for rigorous monitoring of aflatoxins in feed and implementation of mitigation measures to curb the associated feed and food safety risks. Additionally, it is crucial to investigate aflatoxin contamination levels in milk to better understand the potential risks associated with aflatoxin exposure. Overall, these findings indicate significant feed and food safety concerns related to aflatoxins, found in specific locations in this study, that require attention from policy-makers, researchers, administrators, and other concerned bodies.

## 5. Materials and Methods

### 5.1. Description of the Study Area

**Figure 3:**
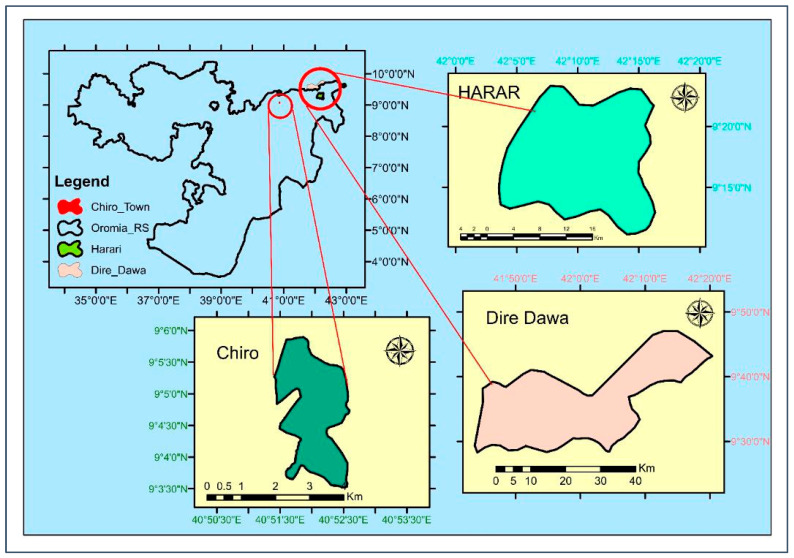
Map showing the location of the study areas: Chiro town (dark green), Dire Dawa city (rose), and Harar city (light green).

Considering their potential for dairy production and their role as the main market centers for milk distribution for the surrounding districts, the three major urban centers of eastern Ethiopia, Dire Dawa city, Chiro town, and Harar city (Figure 3), were specifically selected for this study [38,62,63]. These selected urban centers are situated in different agroecological areas according to elevation. Dire Dawa city is situated in a lowland agroecology with an elevation of 1170 m above sea level and an average annual temperature of 25.2 °C (17.4–33.0 °C), whereas Chiro town has a semiarid agroclimate and is situated at 1757 m above sea level, with an average annual temperature of 20.6 °C (13.3–27.8 °C) [64,65,66]. On the other hand, the majority of Harar city’s land is situated in a midland agroecological location, at 1900–2200 m above sea level, where a few areas are situated in a highland agroecology [67]. Harar city has an annual average temperature of 20.5 °C, ranging from 14.1 to 26.8 °C [66].

### 5.2. Feed Sample Collection

For this study, 180 concentrate feed samples, comprising wheat bran (WB), total mixed ration (TMR), and maize feed (MF), were gathered from two primary feed sources: specialized dairy farms and local markets. Between September 2021 and January 2022, feed samples were gathered from the selected urban centers (i.e., Chiro town, Harar, city and Dire Dawa city). Ten samples of each feed type—MF (10), TMR (10), and WB (10)—were gathered from the specialized dairy farms in each of the identified urban centers. Similarly, ten samples from local markets in each urban center were collected for each of the three feed types: MF (10), TMR (10), and WB (10). As a result, 180 feed samples (10 × 3 × 2 × 3 = 180) were taken and analyzed for aflatoxin contamination.

The sample size was determined using an 86% (0.86) expected prevalence [68] and a 5% level of precision, following the method described by Daniel and Cross [69]. Prior to the collection of feed samples, detailed consultations were held with agricultural administrators, livestock experts, and extension workers in the target urban centers to identify potential kebeles (the smallest administrative unit) for specialized dairy farming and the major feeds. Thus, the livestock development offices were provided a list of specialized dairy farms, from which the sampled dairy farms (which were feeding their cows from MF, TMR, or WB) were randomly selected for feed sample collection. In collaboration with livestock extension workers, local feed retailers or feed shops (selling MF, TMR, or WB) were subsequently selected by visiting the identified marketing centers using systematic random sampling. The feed samples were bought from the identified feed retailers/shops.

Thus, to form an aggregated portion of the sample, a small amount of feed was taken by sampling spears from many locations in different containers/sacks where the lactating cows were being fed. After the samples were well mixed, a 0.5 kg feed sample was collected from the aggregated portion and placed into a labeled sampling bag. The feed samples were subsequently brought to the Dairy Sciences Laboratory of Haramaya University and kept under cool conditions until further analysis was performed. Aflatoxin analysis of the feed samples was performed at the Animal Products, Veterinary Drug and Feeds Quality Assessment Center (APVDFAC) in Addis Ababa.

### 5.3. Feed Sample Extraction

Feed samples were extracted following the procedures outlined in the AOAC official method with slight modifications [70]. Briefly, the feed samples were ground to a particle size of 0.01 mm using the Chincan mill (FW100, Taichung, China). The feed samples were thoroughly mixed to achieve homogeneity. Then, 20 g of each feed sample was weighed into a 500 mL graduated plastic cylinder containing 2 g of sodium chloride and 80 mL of a methanol–water (0.8:0.2 *v*/*v*) solution and shaken using a wrist-action shaker (Wrist-Action Shaker: Model-75, Burrell Company, Sheffield, England) for 30 min. Then, the solutions were filtered into 250 mL glass beakers through a Whatman No. 1 paper.

Next, 20 mL of n-hexane was added to each filtrate, and the solutions were transferred to Falcon test tubes and centrifuged at 2500 rpm for 10 min using the Hermle Labortechnik GmbH, Wehingen, Germany. Following centrifugation, the solutions were added to standing glass beakers for phase separation, and the defatted desiccant was then drained from the supernatant fatty layer of each solution. Subsequently, 7 mL of each defatted solution was mixed with 43 mL of phosphate-buffered saline (PBS) solution and filtered through a 0.22 µm micropore syringe filter. The filtered sample solutions were then subjected to sample clean-up.

### 5.4. IAC Feed Sample Clean-Up

Sample clean-up was performed using the solution obtained from the sample extraction process. Thus, AflaClean immunoaffinity column (IAC) kits (AflaClean LCTech GmbH, Wehingen, Germany) were used to clean up the sample solution for aflatoxins, including AFB_1_, AFB_2_, AFG_1_, and AFG_2_ in feeds. The instructions enclosed with the IAC kits were followed for sample clean-up. Thus, 50 mL of each defatted sample solutions was eluted through IAC kit columns at a rate of 1–2 drops per second using a vacuum pressure pump (Supelco Visiprep™, Darmastadt, Germany). The columns were washed with 10 mL of distilled water at the same flow rate. Subsequently, the bound aflatoxins were eluted with 3 mL of HPLC grade acetonitrile. The eluate was collected in vials and injected into the HPLC for aflatoxins analysis.

### 5.5. HPLC Instrumentation

An Agilent 1260 system (Agilent Technologies, Santa Clara, CA, USA) with a high-performance liquid chromatography (HPLC) instrument coupled with a fluorescence detector (FLD), Chemstation Software (Agilent Technologies), a binary pump, a vacuum degasser, an autosampler, and an Agilent column (Eclipse XDB-C18, 1.8 µm, 4.6 × 50 mm) was used to analyze aflatoxin in the feed samples. The mobile phase was composed of an isocratic mixture of water/acetonitrile/methanol (65:20/15, *v/v*) at a flow rate of 1.4 mL/min. With the column temperature adjusted to 40 °C, the injection volume was set to 20 µL. Fluorescence wavelengths at 365 nm excitation and 435 nm emission were used to detect AFB_1_, AFB_2_, AFG_1_, and AFG_2_ in the feed samples.

### 5.6. Method Validation

Method validation was carried out in compliance with the standards set forth in the AOAC [70]. Consequently, method validation was conducted on the basis of linearity, the limit of detection (LOD), the limit of quantification (LOQ), the percentage of recovery (%R), and the percentage of relative standard deviation (%RSD) (Table 7).

The linearity of the method was determined by preparing calibration curves for each aflatoxin using a standard solution (Sigma-Aldrich^®^ Darmastadt, Germany) at six concentration levels (2–32 µg/kg). The peak areas of aflatoxins against the concentration level of standards were plotted, and linear regression and coefficients of determination (R^2^) were computed. Thus, the coefficients of determination (R^2^) of 0.9997, 0.9998, 0.9999, and 0.9981 for AFB_1_, AFB_2_, AFG_1_, and AFG_2_, respectively, demonstrated linearity.

In addition, the limit of detection (LOD) and limit of quantification (LOQ) were used to demonstrate the method’s sensitivity. Thus, the minimum concentration of an analyte that can be detected was set to be the LOD, whereas the lowest amount of an analyte in a sample that can be quantitatively determined with suitable precision was termed the LOQ. The LOD was calculated as the lowest concentration of the analyte giving a signal response 3 times greater than the average of the baseline noise obtained from ten blank samples (S/N 3:1), and the LOQ was determined as the analyte signal response 10 times greater than the average of the baseline noise obtained from ten blank samples (S/N 10:1). As a result, the limits of detection for AFB_1_, AFB_2_, AFG_1_, and AFG_2_ were 0.54, 0.43, 0.35, and 1.39 µg/kg, respectively. Similarly, the limits of quantification for AFB_1_, AFB_2_, AFG_1_, and AFG_2_ were determined to be 1.65, 1.30, 1.07, and 4.20 µg/kg, respectively.

Furthermore, the accuracy and precision of the method were evaluated by spiking samples at six known aflatoxin concentrations in triplicate (Table 7). The method’s repeatability was measured using the percent of recovery (%R), whereas precision was assessed using the percent of relative standard deviation (%RSD), as described in the following equations:

(1) $$\%R=\frac{Conc.\;in\;spiked\;sample\;-\;Conc.\;in\;unspiked\;sample}{Conc.\;in\;the\;unspiked\;sample}\times 100\%$$

(2) $$\%RSD=\frac{SD\;of\;replicated\;concentration}{Mean\;of\;\%R}\times 100\%$$

Accordingly, the %R ranged from 72.0 to 97.28%, 75.9 to 90.10%, 73.2 to 114.79%, and 70.45 to 100.24% for AFB_1_, AFB_2_, AFG_1_, and AFG_2_, respectively. This demonstrates that the analytical method was accurate, as the percentages of recovery were within the range of 70–120% as specified in the AOAC [70]. Similarly, ranges of 0.11% to 0.85% of the percentage relative standard deviation of 0.21%, 0.15%, 0.11%, and 0.57% for AFB_1_, AFB_2_, AFG_1_, and AFG_2_, respectively, were found. The %RSD of this investigation demonstrated that the method is precise as the values were less than 5%.

### 5.7. Identification of Aflatoxigenic *Aspergillus* Species

To screen for aflatoxigenic *Aspergillus* species, 1 g of a ground feed sample was added to a sterilized test tube along with 9 mL of distilled water. The mixture was then vortexed for 5 min [71]. Next, the serial dilution technique was employed, with dilutions of 10^−1^, 10^−2^, 10^−3^, and 10^−4^ [72]. From each dilution, 1 mL of the solution was dispensed into a 90 mm Petri dish containing *Aspergillus flavus* and *Parasiticus* Agar (AFPA) to isolate and identify *Aspergillus* species from the feed samples. The AFPA medium was prepared from 20 g/L yeast extract, 10 g/L bacteriological peptone, 0.5 g/L ferric ammonium citrate, and 15 g/L agar [3].

Furthermore, the technique described by Ahmed et al. [73] and Abd El-Aziz et al. [74] was employed to assess the aflatoxigenic potential of *Aspergillus* species colonies by coconut agar medium (CAM) using UV fluorescence UVITEC, Cambridge, UK at a wavelength of 365 nm. To obtain pure cultures and screen for their aflatoxigenic potential colonies of *A. flavus* and *A. parasiticus* species grown on AFPA were sub-cultured onto CAM and incubated at 26 ℃ in the dark for 5–7 days. Accordingly, the colonies that produced aflatoxin exhibited blue-green fluorescence, whereas the colonies that did not produce aflatoxin did not exhibit such fluorescence.

### 5.8. Statistical Analysis

The data collected were checked and entered into Microsoft Excel 2016 (MS Excel^®^) and then exported to SAS v9.4 software (SAS Institute, Cary, NC, USA) for analysis. The occurrence of aflatoxins in dairy feed samples from different study sites, feed sources, and feed types was analyzed and presented in graphs and frequency tables. For the mean concentration of aflatoxin in the feed samples, analysis of variance (ANOVA) was performed to compare their differences across the study sites, feed sources, and feed types. Meanwhile, Duncan’s test was used to determine the difference in the mean concentration (µg/kg) of each aflatoxin in the feed samples. Furthermore, aflatoxigenic *Aspergillus* species were correlated with aflatoxin levels in feed.

## Figures and Tables

**Table 1:** Frequency of occurrence and level of TAF in feed across study sites, feed sources, and feed types.

Categories		N	^1^ Fr. (%)	^2^ Level of TAF (µg/kg)		
				Mean ± SE	Minimum	Maximum
Study sites						
	Chiro	60	47 (26.1)	33.60 ± 8.2 ^a^	ND	150.6
	Dire Dawa	60	53 (29.4)	86.93 ± 8.2 ^b^	ND	455.8
	Harar	60	49 (27.2)	41.50 ± 8.2 ^a^	ND	229.2
	DF			2		
	MS		ns	49,728.62 **		
Feed sources						
	Dairy farms	90	75 (41.7)	55.7 ± 6.7	ND	455.8
	Local markets	90	74 (41.1)	52.2 ± 6.7	ND	245.6
	DF			1		
	MS		ns	557.22 ns		
Feed types						
	Maize feed	60	50 (27.8)	43.8 ± 8.2 ^a^	ND	175.9
	Total mixed ration	60	54 (30.0)	92.2 ± 8.2 ^b^	ND	455.8
	Wheat bran	60	45 (25.0)	26.0 ± 8.2 ^a^	ND	186.2
	DF			2		
	MS		ns	70,298.75 **		

Column values with different superscript letters are significantly different (α = 0.05); ns = *p* > 0.05; ** = *p* < 0.01; N = total number of samples analyzed; ^1^ Fr. (%) = frequency of occurrence; ^2^ TAF = AFB_1_ + AFB_2_ + AFG_1_ + AFG_2_; MS = mean square; DF = degree of freedom; ND = not detected.

**Table 2:** Level of AFB_1_ and AFB_2_ in feeds across study sites, feed sources, and feed types.

Categories		N	AFB_1_			AFB_2_		
			^1^ Fr. (%)	M ± SE (µg/kg)	Range (µg/kg)	^1^ Fr. (%)	M ± SE (µg/kg)	Range (µg/kg)
Study sites								
	Chiro	60	22.3 ^a^	18.40 ± 5.3 ^a^	LOD–94.80	21.1	1.63 ± 0.6 ^a^	LOD–14.1
	Dire Dawa	60	29.4 ^b^	43.98 ± 5.3 ^b^	LOD–303.5	25.0	5.69 ± 0.6 ^b^	LOD–30.3
	Harar	60	20.7 ^a^	22.06 ± 5.3 ^a^	LOD–182.5	23.3	2.55 ± 0.6 ^a^	LOD–15.4
	DF			2			2	
	MS		*	11,480.7 ***		ns	271.4 **	
Feed sources								
	Dairy farms	90	37.2	28.81 ± 4.2	LOD–303.5	37.2	3.87 ± 0.5	LOD–30.3
	Local markets	90	35.0	27.48 ± 4.2	LOD–128.4	32.2	2.71 ± 0.5	LOD–18.0
	DF			1			1	
	MS		ns	78.1 ns		ns	59.9 ns	
Feed types								
	Maize feed	60	21.3 ^b^	22.08 ± 5.2 ^a^	LOD–94.8	21.7	2.42 ± 0.6 ^a^	LOD–11.4
	Total mixed ration	60	30.7 ^a^	50.67 ± 5.2 ^b^	LOD–303.5	26.1	4.74 ± 0.6 ^b^	LOD–25.3
	Wheat bran	60	20.2 ^b^	11.69 ± 5.2 ^a^	LOD–77.0	21.7	2.71 ± 0.6 ^a^	LOD–30.3
	DF			2			2	
	MS		*	24,455.1 **		ns	96.2 **	
SS*FT				*			ns	

Column values under the same category with different superscript letters are significantly different (α = 0.05); ns = *p* > 0.05; * = *p* < 0.05; ** = *p* < 0.01; *** = *p* < 0.001; N = total number of samples examined; M = mean; ^1^ Fr. (%) = frequency of occurrence; SE = standard error of mean; SS = study sites; FT = feed types; limit of detection (LOD) is ≤0.54 µg/kg for AFB_1_; LOD is ≤0.43 µg/kg for AFB_2_.

**Table 3:** Interaction between concentration of AFB_1_ and AFG_1_ and feed types and study sites.

Study Sites	Feed Types	N	Types of Aflatoxins					
			AFB_1_			AFG_1_		
			M ± SE (µg/kg)	95% Confidence Interval		M ± SE (µg/kg)	95% Confidence Interval	
				Lower	Upper		Lower	Upper
Harar	Maize feed	60	16.9 ± 8.9 ^a^	−0.8	34.7	14.7 ± 4.6 ^a^	5.5	23.9
	Total mixed ration	60	38.4 ± 8.9 ^b^	20.6	56.2	24.3 ± 4.6 ^b^	15.1	33.5
	Wheat bran	60	10.8 ± 8.9 ^a^	−6.9	28.6	7.4 ± 4.6 ^a^	−1.7	16.6
Dire Dawa	Maize feed	60	19.5 ± 8.9 ^a^	1.7	37.2	21.1 ± 4.6 ^a^	11.9	30.3
	Total mixed ration	60	94.9 ± 8.9 ^b^	77.1	112.6	60.0 ± 4.6 ^b^	50.8	69.2
	Wheat bran	60	17.5 ± 8.9 ^a^	−0.2	35.3	15.6 ± 4.6 ^a^	6.4	24.8
Chiro	Maize feed	60	29.8 ± 8.9 ^b^	12.0	47.5	14.3 ± 4.6 ^a^	5.1	23.5
	Total mixed ration	60	18.7 ± 8.9 ^b^	0.9	36.4	14.2 ± 4.6 ^a^	5.0	23.4
	Wheat bran	60	6.6 ± 8.9 ^a^	−11.0	24.4	7.0 ± 4.6 ^a^	−2.2	16.2
DF			4			4		
MS			10,664.9 ***			2618.052 ***		

Column mean values under the same category that bear different superscript letters are significantly different; *** = *p* < 0.001; M = mean; SE = standard error of mean; DF = degree of freedom; MS = mean square.

**Table 4:** Level of AFG_1_ and AFG_2_ in dairy feeds across study sites, feed sources, and feed types.

Categories		N	AFG_1_			AFG_2_		
			^1^ Fr. (%)	M ± SE (µg/kg)	Range (µg/kg)	^1^ Fr. (%)	M ± SE (µg/kg)	Range (µg/kg)
Study sites								
	Chiro	60	22.8 ^a^	11.87 ± 2.7 ^a^	LOD–49.60	20.6	1.67 ± 0.5 ^a^	LOD–9.40
	Dire Dawa	60	28.3 ^b^	32.25 ± 2.7 ^b^	LOD–125.5	25.6	5.01 ± 0.5 ^b^	LOD–21.5
	Harar	60	20.0 ^a^	15.50 ± 2.7 ^a^	LOD–83.70	22.2	1.37 ± 0.5 ^a^	LOD–9.80
	DF			2			2	
	MS		*	7086.2 ***		ns	244.3 **	
Feed sources								
	Dairy farms	90	36.1	19.48 ± 2.2	LOD–125.5	35.6	3.60 ± 0.4	LOD–21.5
	Local markets	90	35.0	20.26 ± 2.2	LOD–97.30	32.2	1.77 ± 0.4	LOD–12.7
	DF			1			1	
	MS		ns	27.3 ns		ns	150.2 ns	
Feed types								
	Maize feed	60	21.9 ^a^	16.75 ± 2.6 ^a^	LOD–75.40	22.2	2.60 ± 0.5 ^a^	LOD–11.5
	Total mixed ration	60	28.7 ^b^	32.87 ± 2.6 ^b^	LOD–125.5	23.3	3.86 ± 0.5 ^b^	LOD–21.5
	Wheat bran	60	20.7 ^a^	10.00 ± 2.6 ^a^	LOD–57.40	21.7	1.58 ± 0.5 ^a^	LOD–21.5
	DF			2			2	
	MS		*	8280.0 **		ns	78.5 **	
SS*FT				**			ns	

Column mean values under the same category that bear different superscript letters are significantly different from each other; ns = *p* > 0.05; * = *p* < 0.05; ** = *p* < 0.01; *** = *p* < 0.001; N = total number of samples examined; M = mean; ^1^ Fr. (%) = frequency of occurrence; SE = standard error of mean; SS = study sites; FT = feed types; limit of detection (LOD) is ≤0.35 µg/kg for AFG_1_; LOD is ≤1.39 µg/kg for AFG_2._

**Table 5:** Feed samples exceeding the FDA/ESA regulations limit for AFB_1_ and TAF.

Categories		N	Samples Exceeding FDA/ESA for AFB_1_ (20 µg/kg)		Samples Exceeding ESA for TAF (40 µg/kg)	
			n	Fr. (%)	n	Fr. (%)
Study sites						
	Chiro	60	15	25.0	16	26.7
	Dire Dawa	60	25	41.7	34	56.7
	Harar	60	20	33.3	20	33.3
Feed sources						
	Dairy farms	90	38	42.2	39	43.3
	Local markets	90	22	24.4	31	34.4
Feed types						
	Maize feed	60	20	33.3	26	43.3
	Total mixed ration	60	30	50.0	32	53.3
	Wheat bran	60	10	16.7	12	20.0
Overall		180	60	33.3	70	38.9

N = number of samples examined; n = number of samples with AFB_1_ or TAF exceeding the FDA/ESA regulation limit; Fr. (%) = frequency of samples exceeding the FDA/ESA regulation limit (n/N).

**Table 6:** Correlation of aflatoxigenic *A. flavus* and *A. parasiticus* species with the levels of aflatoxins in feed samples.

Aflatoxigenic Counts	AFB_1_	AFB_2_	AFG_1_	AFG_2_
*A. flavus*	0.47 **	0.46 **	-	-
*A. parasiticus*	0.43 **	0.40 **	0.50 **	0.39 **

** = *p* < 0.01.

**Table 7:** Method performance of aflatoxin analysis in feed samples.

Aflatoxins	Spike (ppb)	LOD (µg/kg)	LOQ (µg/kg)	Linearity Equation	%R (%)	%RSD (%)
AFB_1_	2–32	0.54	1.65	Y = 1.0691x − 1.5631	72.0–97.28	0.21
AFB_2_	2–32	0.43	1.30	Y = 1.1438x − 1.6437	75.9–90.10	0.15
AFG_1_	2–32	0.35	1.07	Y = 1.2482x − 1.3313	73.2–114.79	0.11
AFG_2_	2–32	1.39	4.20	Y = 1.1888x − 2.6024	70.45–100.24	0.57

## Data Availability

The raw data supporting the conclusions of this article will be made available by the authors upon request.
